# Can we predict the altered subjective quality assessment of sound after ear canal surgery?

**DOI:** 10.1007/s00405-020-05971-3

**Published:** 2020-04-25

**Authors:** M. R. Zwemstra, P. Brienesse, F. A. Ebbens, M. J. F. de Wolf, E. van Spronsen

**Affiliations:** Department of Otorhinolaryngology, Academic Medical Center, Meibergdreef 9, 1105 AZ Amsterdam, The Netherlands

**Keywords:** Ear canal, Acoustics, Sound quality

## Abstract

**Purpose:**

To correlate the subjective quality assessment of ear canal acoustics of the participants to the objective measurement of the ear canal acoustics. The objective ear canal acoustics is the frequency-dependent modulation of soundwaves through the ear canal. Our second objective is to design a model to predict the subjective quality of sound based on the altered objective ear canal acoustics.

**Methods:**

To determine the frequency-dependent modulation of the soundwaves the real-ear unaided gain (REUG) of the ear canal is measured. 40 participants with normal hearing were presented six simulated sound fragments representing the acoustic properties of six different ear canals (REUG). These six sound fragments were built based on the difference between these six REUGs and the average REUG of a normal adult ear canal. Subjective sound quality was evaluated using a *VAS* score and a paired comparison score.

**Results:**

We found a strong correlation between the objective ear canal acoustics and the subjective assessment of the quality of sound (Spearman’s rho—0.89). Our linear mixed *VAS* model for individual participants has an intercept of 95.6 and a slope of − 4.2 (*p* < 0.001). The paired comparison analysis endorsed our findings that an increased difference in REUG is predictive for a decreased quality assessment of ear canal acoustics.

**Conclusion:**

There is a strong correlation between the subjective evaluation of ear canal acoustics and the objective quality assessment of ear canal acoustics. Our models show that an increased difference in REUG predicts a decreased quality of ear canal acoustics.

## Introduction

Acoustics is defined as the science that deals with the production, control, transmission, reception, and effect of the sound [1]. External ear acoustics is the modulation of sound in the ear canal. The external auditory ear canal transfers soundwaves from the concha to the eardrum and acts as a resonant tube [2]. Due to its anatomical dimensions the external auditory ear canal acts as a filter to reduce low frequencies and enhance mid to high frequencies [3]. Surgical modification of the osseous external auditory canal (OEAC) changes the acoustic properties of the external ear canal [4–9]. For example, in case of a cavity condition, the acoustic properties shift towards an amplification of the soundwaves of low to mid frequencies and an reduction in soundwaves of high frequencies [8]. In case of revision, radical cavity surgery with reconstruction of the posterior wall of the cavity near-normal acoustic characteristics were measured [9]. Even less extensive surgical alterations of the OEAC, such as an osseous canalplasty, lead to changes in ear canal acoustics [9]. These observations seem to indicate that volume changes of the ear canal lead to alterations in ear canal acoustics [8, 9]. In previous studies, we have demonstrated that these surgical alterations of the OEAC provoke an altered perception of the sound [8, 9]. In patients with hearing loss and hearing aids, we know that altered acoustics result in a reduced quality of life [10, 11]. For clinicians, it would be very helpful to be able to inform patients pre-operatively to what extend their ear canal acoustics changes after surgery of the ear canal. Therefore, we have to assess to what extent patients are able to recognize differences in ear canal acoustics, by investigating the relation between the objective measurement and subjective experience of the acoustics of an altered ear canal. Although a correlation between subjective sound quality assessment and objective acoustic quality assessment in Portuguese churches has been described earlier [12], to our knowledge we are the first to investigate the correlation between objective measurements of external auditory canal acoustics and subjective quality assessment of ear canal acoustics.

The primary objective of this study is to correlate subjective quality assessment of acoustics to the objective measurement of external ear canal acoustics. Our second objective is to design a model to predict the subjective quality of sound based on the altered objective ear canal acoustics.

## Participants and methods

### Subjects

We included 40 individuals with normal hearing. Of these 40 individuals, 27 (67.5%) were female and 13 (32.5%) were male. The average age of all participants was 31.6 years (median 28, range 21–73 years). Pure tone hearing thresholds were 20 dB HL or better at 0.25, 0.5, 1, 2, 4 and 8 kHz. All participants were healthy and had no history of ear disease. All participants agreed to participate in the study. The study protocol was in accordance with the Helsinki declaration and was approved by the ethical review board. None of the authors had a conflict of interest.

### Methods

A detailed description of our methods has been reported previously [9]. In summary, we have created six filtered sound fragments that simulate six different acoustic properties based on the REUG of six different ear canals. These filtered sound fragments are assessed by 40 participants on subjective quality of sound assessment using *VAS* scores and paired comparison.

To define six filters we measured acoustic properties of six ear canals, via the Real Ear Unaided Gain (REUG), being the frequency-dependent gain in decibels (dB) of the soundwave from concha to eardrum [14]. The REUG is measured using a microphone inserted in the ear canal that measures the frequency-dependent gain of the soundwave in decibels of a well-defined broadband sound stimulus from outside the ear canal. Of these six ear canals, five ear canals were cavities after canal wall down surgery (more than 15 years ago) and one normal shaped ear canal. All ear canals were dry and properly cleaned before REUG measurement.

### Simulation of the acoustic properties of six individual ear canals

The acoustic properties of the ear canal can be characterized by measuring the REUG [14]. Differences between individual REUGs represent differences in acoustic properties of individual ear canals. The acoustic effect of the measured acoustic properties of the ear canals can be simulated in the participant’s ear canal by filtering the incoming sound stimulus. Therefore, we use the difference between the REUG of an average normal ear canal and the REUG of the measured ear canal, using the REM module of the Affinity 2.0 Hearing Aid Analyzer platform (Interacoustics, Denmark). For our participants, this filtering results in the same distribution of sound pressure (acoustics) at the eardrum as in the original ear canal, thereby mimicking the acoustic effect of e.g., a cavity in a normal ear [8, 9]. The REUG of the average, normal adult ear canal is derived from de data by Dillon [14], also used as a reference in the REM module of the Affinity 2.0 Hearing Aid Analyzer. The discrete numbers given by Dillon for the (half) octave frequencies were interpolated for the intermediate frequency values using the Cubic Spline interpolation function (all computing was done using Matlab version R2016b). This resulted in an interpolated mean REUG of a normal adult ear canal, in short denoted as the ‘Dillon line’.

**Fig. 1:**
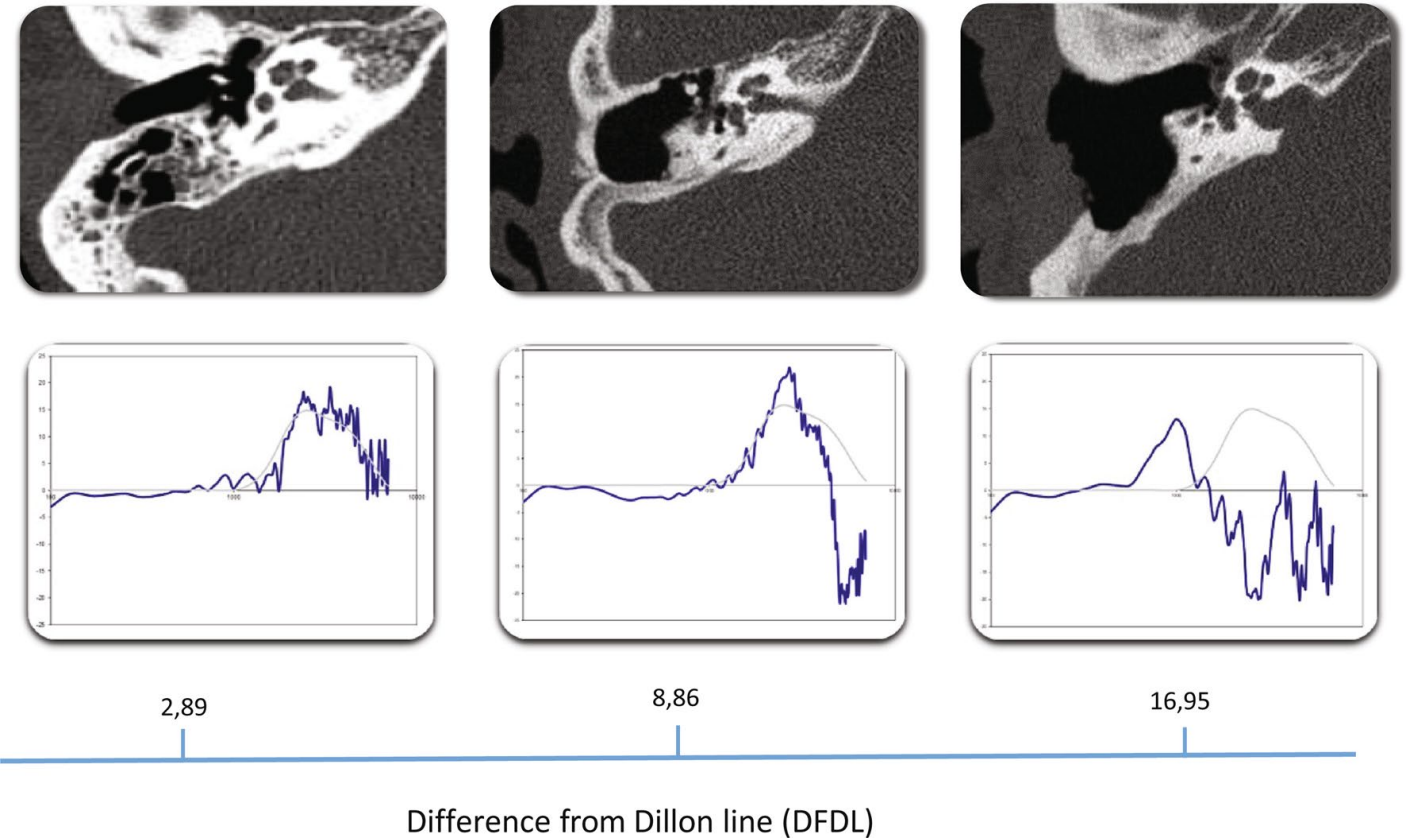
This figure illustrates the Difference from Dillon line (DFDL). The three figures below represent the REUG of the corresponding CT-scan of, respectively, a normal ear canal, a small cavity and a large cavity. The blue line is the actual REUG as it is measured in the corresponding ear canal, the grey line is the mean REUG of a normal adult ear canal (refered to as the Dillon line). The difference between these two lines is used to construct an acoustic filter for the stimuli presented to the participants. The DFDL represents the total area between the blue and the grey line, as a measure of the mismatch of an individual REUG from the normal situation

We made recordings of Dutch speech (two male and two female speaker sentences based on the VU98 sentence material (Versfeld et al. [13]), filtered to simulate the acoustic properties of five cavities after canal wall down surgery and one ‘normal’ ear canal. The REUG of the five cavities after canal wall down surgery and one normal ear canal were measured using the REM module of the Affinity 2.0 Hearing Aid Analyzer platform (Interacoustics, Denmark). Six filters c.q. simulated conditions were built on the differences between these six individual REUGs and the average REUG of a normal adult ear canal, the Dillon line (see Fig. 1). The seventh ‘reference’ condition consisted of the unfiltered speech material.

The extend to how the individual REUGs differ from the Dillon line is denoted as the DFDL (difference from Dillon line). This DFDL is the root mean square (RMS) value of the difference between the individual REUG and the Dillon line at all of the (185) discrete frequencies ranging from 125 to 8000 Hz. The five cavity ear canals had a DFDL ranging from 6.04 to 16.95. The normal ear canal used as one of the six filtered conditions had a DFDL of 2.89 (Fig. 1).

### Perceptual evaluation

A detailed description of the perceptual evaluation was reported previously [8]. The perceptual evaluation experiment was performed with a paired comparison category rating between two fragments (‘A’ and ‘B’), according to ITU-T 1996 [15]. Participants were asked which fragment sounded the most natural using a seven point scale. Each filtered condition was compared to the unfiltered reference condition. Fragments of filtered conditions were based on the six conditions (five cavities and one ‘normal’ ear canal) previously described. All conditions were presented by two male and two female voices and were measured twice: one time using the filtered sentence as ‘A’ and the reference sentence as ‘B’, and one time in a reversed fashion. Thus, 48 paired comparisons, together with four control comparisons in which the seventh unfiltered condition was compared to itself, making a total of 52 paired comparisons were presented in random order.

The paired comparison category rating task was followed by a *VAS* score task, evaluating the ‘overall’ sound quality of the seven conditions, zero being the worst possible outcome and 100 the best. Again, the seven conditions were presented in random order by playing four different Dutch sentences.

All of the speech material was presented in free field at a level of 65 dB(A), using a loudspeaker in front of the listener (0^°^ angle).

### Statistical analysis

Data are expressed as numbers. Preliminary data analysis was performed using box-plots for the paired comparisons and scatter plots for the *VAS* scores. Correlation on group level was measured using Spearman’s rho.

A mixed linear model was used to predict and relate the effect of the DFDL, expressed as a sample of a continuous measure, to the *VAS* score for the individual expressed as a continuous measure from 0 to 100 in SPSS (IBM SPSS Statistics for Windows, Version 25.0. Armonk, NY: IBM Corp). We compared three models to investigate what model would be the most realistic model for our data. In our first model, the *VAS* score was modeled with fixed intercept and slope as well as a random intercept and a random slope to account for baseline differences as well as differences in slope between participants. In our second model, we estimated the same parameters but excluded the data that were obtained from the theoretical Dillon line. In preliminary analysis saturation of *VAS* scores was reached at this point. In our third model, we used a quadratic mixed model: we estimated a fixed and random intercept and slope, and a random quadratic slope. We transformed our Beta to a odds ratio to evaluate the prognostic effect of the models.

To analyze the paired comparisons (categorical data) we used a cumulative mixed linear model rating using the ‘ordinal’ package in R [16]. The paired comparison measurements are stated on a seven-point scale ranging from + 3 (the filtered sentence sounds much more natural than the reference) to − 3 (the reference sentence sounds much more natural than the filtered sentence). A score of zero means there is no difference noticeable in the naturalness of sound. We included random effects for participants (*n* = 40) and sentence number (*n* = 4). Gender of the reader of the sound fragment, the condition (DFDL) and, whether the condition was compared for the first or the second time, were estimated as fixed effects.

## Results

**Fig. 2:**
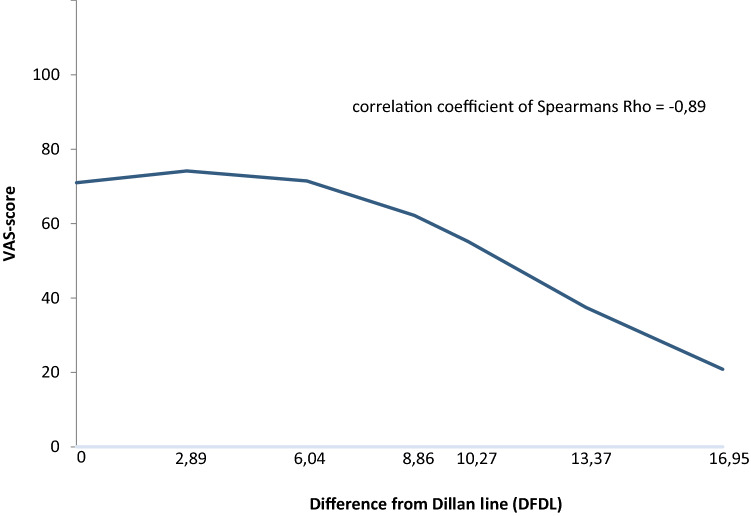
In this figure, the mean *VAS*-score of all participants is shown. Using Spearman’s Rho we found a correlation coefficient of − 0.89, indicating a strong correlation

**Fig. 3:**
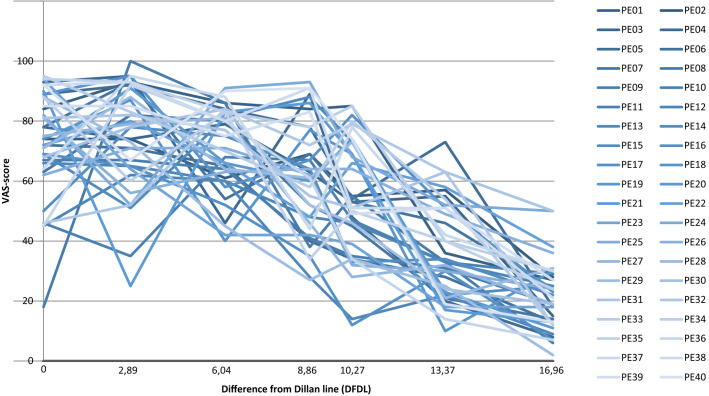
In this figure, we demonstrate the *VAS*-scores and the course of the individual line of all participants

An overview of the mean *VAS* score of all participants together is shown in Fig. 2. Using Spearman’s rho we found a correlation coefficient of − 0.89 (*p* = 0,003). An overview of the *VAS* from all participants is shown in Fig. 3. We can clearly see that there is a difference between *VAS* scores of the individuals. Therefore, we built prognostic models to predict the outcome for the individual participant.

An overview of these prognostic models for the individual participant is seen in Table 1. In our first linear mixed *VAS* model (I) we found a significant correlation (*p* < 0.001) with an intercept of 84.6 (SD 1.4) and a slope (Beta) of − 3.4 (SD 0.43). The odds ratio was 0.035. This means for every increase in difference from the Dillon line the odds are 0.035 that the subjective sound quality decreases.

**Table 1:** In this table, we demonstrate the results of the models

	Mean		*p* value		Standard deviation		Odds ratio
Model I							
Intercept	84.6		< 0.001		± 1.4		0.035
Slope	− 3.4		< 0.001		± 0.43		
Model II							
Intercept	95.6		< 0.001		± 1.60		0.014
Slope	− 4.2		< 0.001		± 0.46		
Model III							
Intercept	73.9		< 0.001		± 1.38		0.108
Slope	− 0.2		< 0.001		± 0.15		

Model I. Linear mixed model with fixed and random intercept and slope. *Y* = (slope × delta Dillon) + intercept. Model II. Linear mixed model with fixed and random intercept and slope with the theoretical Dillon REUG excluded. *VAS* = (slope × delta Dillon) + intercept. Model III. Quadratic mixed model with fixed and random intercept and slope. *Y* = (slope × delta Dillon × delta Dillon) + intercept

**Fig. 4:**
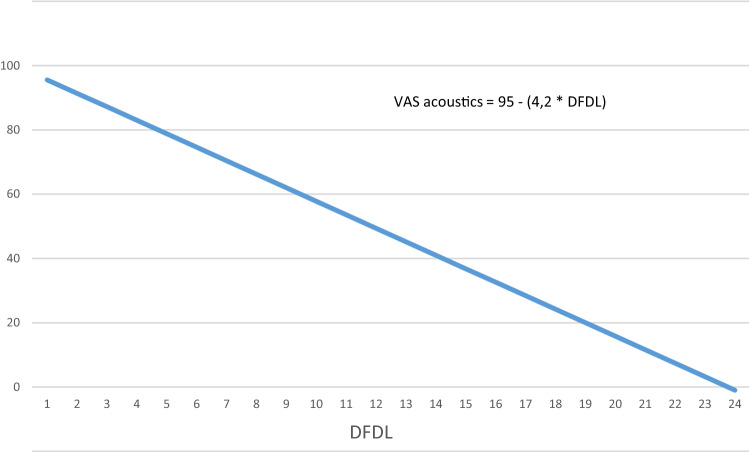
This figure represents the prognostic value of the DFDL on subjective quality assessment for the individual. An increasing DFDL has a negative of 4.2 (SD ± 0.46). The odds risk is 0.014. This means for every increase in DFDL the chance is 0.014 this will lead to an increase in subjective quality assessment of sound, representing a highly unlikely outcome to happen

In our second linear mixed *VAS* model (II) (the reference Dillon line was excluded) we found a significant correlation (*p* < 0.001) with an intercept of 95.6 (SD 1.60) and a slope (Beta) of − 4.2. (SD 0.46) (see Fig. 4). The odds ratio was 0.014.

In the quadratic mixed *VAS* model (III) we found a significant correlation (*p* < 0.001) with an intercept of 73.9 (SD 1.38) and a slope (Beta) of − 0,2 (SD 0.15). The odds ratio was 0.108.

**Fig. 5:**
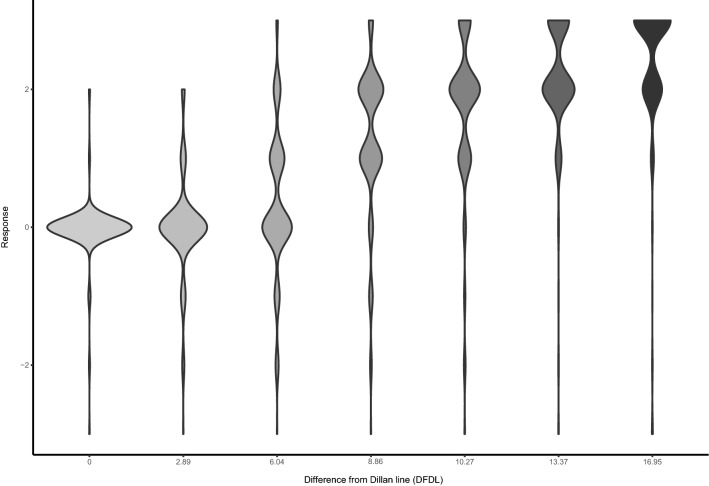
In this violin plot, the paired comparison measurements are displayed. They are stated on a seven-point scale ranging from + 3 (the reference sentence sounds much more natural than the filtered sentence) to − 3 (the reference sentence sounds much more natural than the filtered sentence). A score of zero means there is no difference noticeable in the naturalness of sound. The size of the belly of the violin plot is indicative for the density of response on naturalness

Our cumulative mixed linear odds ratio of difference from the Dillon line is 1.48 (B 0.39 SD ± 0.11, *p* < 0.001). This implies that every step (on the seven-point scale) away from the Dillon line the odds are 1.48 the participant judges the sound as less natural. Figure 5 shows the paired comparison responses of all participants in a violin plot. Clearly one can see that the distribution of responses shifts toward less natural in comparison to the reference sound as the DFDL increases. The effect of gender in sound (OR 0.93 B − 0.07) was not significant. The effect of repetition (i.e. the first or second time comparing the same conditions) was significant (OR 0.76, B − 0.28, *p* < 0.001). This shows that when a repeated measurement is done the odds are 0.76 that the sound will be perceived to be less natural.

## Discussion

To our knowledge, we are the first to associate the subjective quality assessment of ear canal acoustics to the objective measurement of external ear canal acoustics. We have previously demonstrated that surgery of the osseous external ear canal leads to a deterioration in the objective measurement of acoustics [8, 9]. With our current results, we demonstrated a strong correlation (Rho − 0.89) between objective quality assessment of acoustics and subjective assessment of the quality of sound (see Fig. 2). For the group, we now know there is a strong correlation.

Our second objective was to design a model which predicts the effect of an alteration of the acoustics of the OEAC for individual patients. Therefore we investigated three models (see Table 1). In all three models, we have demonstrated that an increased DFDL is predictive of a decreased subjective perception of ear canal acoustics, measured with a *VAS* score.

In our models, the intercept represents the subjective quality assessment of the Dillon line. In our first model, we observed that participants did not judge the Dillon line with a *VAS* score of 100. Instead, saturation was reached around a *VAS* score of 85. This phenomenon has previously been described as end-aversion bias [17]. It refers to the reluctance of some respondents to use the extreme portions of a *VAS*-scale. When we excluded the reference sentences from our current analysis we found (model II) an intercept of 95. Thereby bypassing the end-aversion bias. The quadratic model was not suitable for our data since it corresponds more to a linear relationship. The linear model with excluded Dillon sentences enables us to predict what the effect of surgery of the OEAC on subjective ear canal acoustics will be (see Fig. 4).

With our paired comparison measurement we used a more natural way of comparing ear canal acoustics. The correlation and predictive value of an increased DFDL for a decreased subjective assessment of naturalness of sound measured with paired comparisons endorsed our findings with the *VAS* score. These findings support our previous finding that altered ear canal acoustics is predictive for a decreased subjective assessment of the quality of sound [18].

The effect of repetition of the comparison had an odds ratio of 0.76. This indicates a chance of 0.76 that the repeated sentence is judged to be less natural. An odds ratio of close to one is indicative of a low prognostic effect. In social sciences, this effect is known as the priming effect [19].

All of our participants had good hearing and normal ear canals. They were able to detect and quantify differences in ear canal acoustics. It is unclear whether patients with altered ear canal acoustics (e.g., after canal wall down mastoidectomy) still suffer from changed sound quality after years. Possibly there is habituation. Further research should investigate this possible effect.

In our current study, we demonstrate a linear model where an increased DFDL is predictive for a decreased subjective assessment of the ear canal acoustics. We have previously demonstrated that an altered anatomy of the ear canal is correlated to an impaired assessment of the quality of sound [8, 9]. With these results, we are able to do additional research to the acoustic effects of surgery of the ear canal. Future prospective research with this model enables us to empower our model.

As otolaryngologists we aim to improve the quality of life of our patients. In patients with hearing loss and hearing aids, reduced hearing and altered ear canal acoustics result in a reduced quality of life [10, 11]. In the near future, we hope to inform and predict the improvement of ear canal acoustics with ear canal surgery to improve the quality of life of our patients.

## Conclusion

There is a strong correlation between the subjective evaluation of ear canal acoustics and the objective quality assessment of ear canal acoustics. Our models show that an increased DFDL is predictive for a decreased quality assessment of the ear canal acoustics.
